# The effects of facial appearance on measures of generalized trust

**DOI:** 10.1038/s41598-024-69562-6

**Published:** 2024-08-08

**Authors:** Blaine G. Robbins, Maria S. Grigoryeva

**Affiliations:** Division of Social Science, New York University Abu Dhabi, PO Box 129188, Abu Dhabi, United Arab Emirates

**Keywords:** Psychology, Human behaviour

## Abstract

Research suggests various associations between generalized trust and a wide range of economic, political, and social dimensions. Despite its importance, there is considerable debate about how best to measure generalized trust. One recent solution operationalizes generalized trust as the average of trust ratings across a small set of trust domains and human faces. Here, we investigate whether heterogeneity in facial appearance affects the psychometric properties of these new instruments. In a survey experiment conducted with a sample of U.S. adults (*n* = 5001), we randomly assigned respondents to one of five conditions that varied the features of human and AI-synthesized faces. Irrespective of the condition, respondents rated each face along four trust domains. We find that facial heterogeneity has negligible effects on the measurement validity and measurement equivalence of these new instruments. Small differences are observed on a subset of faces for some psychometric tests. These findings contribute to a growing body of work using faces to measure generalized trust, and demonstrate the utility of using AI-synthesized faces in social science research more broadly.

## Introduction

Generalized trust, or default expectations about the trustworthiness of people in general, is the foundation of well-functioning communities, markets, and hierarchies^1–3^. Despite long-standing interest in generalized trust, the concept has proven difficult to measure: research consistently shows that common measures of generalized trust are inaccurate and non-invariant^4–10^. This is because the terms used in traditional measures, such as the “most-people trust” question, which in its classic form asks, “Some people say that most people can be trusted. Others say you can’t be too careful in your dealings with people. How do you feel about it?” means different things to different people^6,9,11–13^. In particular, the literature shows that interpretations of “most people”^6,11–13^ and what “people can be trusted” to do^14,15^ vary from one respondent to the next, leading to measurement error and biased responses.

To address these issues, Robbins^7^ recently developed a new instrument—the Stranger Face Trust scale (SFT)—that measures generalized trust as the average of trust ratings across a small set of trust domains and human faces. By presenting respondents with a standardized set of specific strangers and particular matters, Robbins^9,10^ finds that the measurement equivalence problems plaguing traditional measures of generalized trust are mitigated with SFT. While various psychometric tests also provide strong empirical support for the reliability and validity of SFT^7–10^, there is little evidence on whether facial appearance biases previous tests. Assessing facial heterogeneity is important because, if SFT is valid and reliable across different sets of faces, it would indicate that SFT accurately measures generalized trust regardless of the faces being assessed, and that SFT can be modified for different settings without sacrificing validity and measurement equivalence.

**Figure 1:**
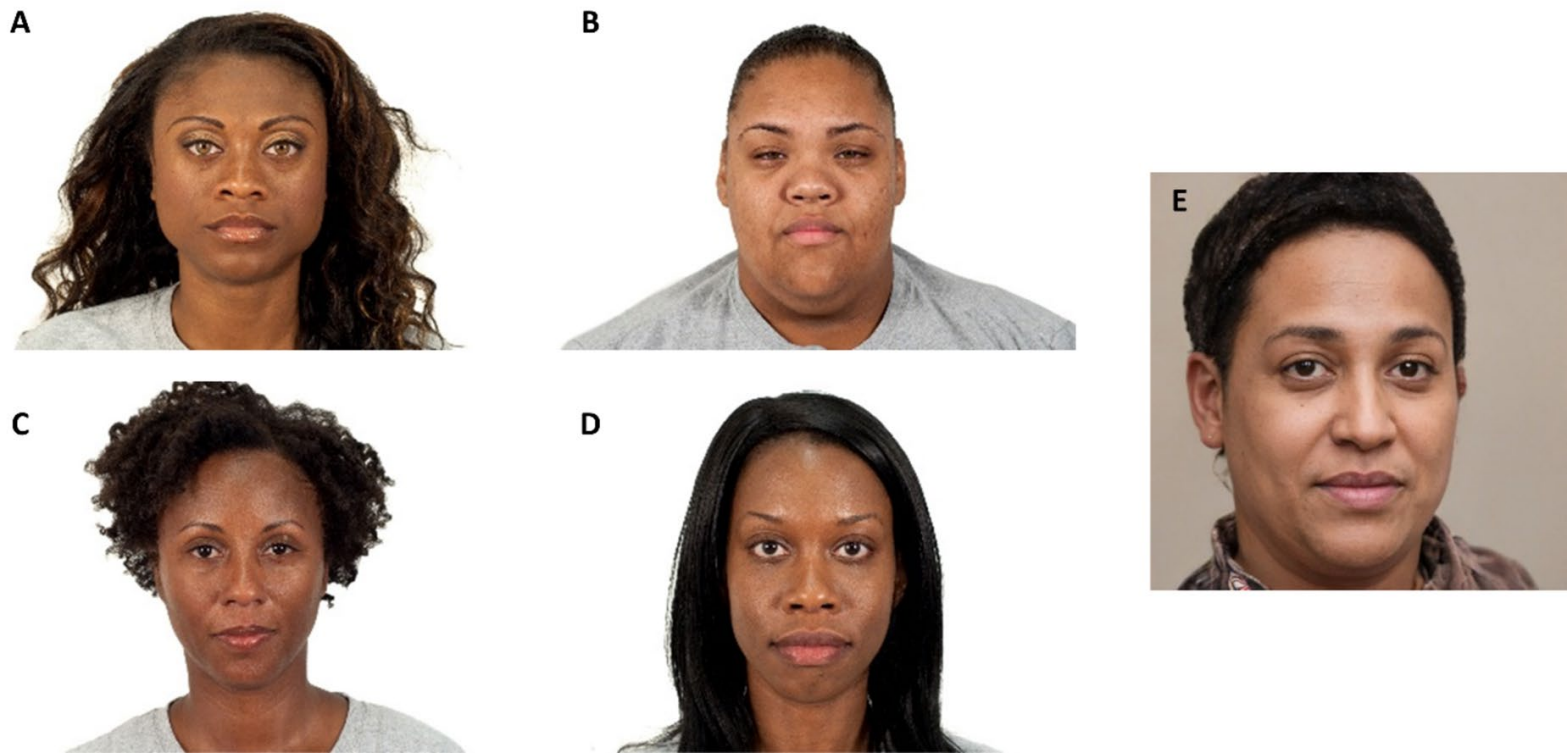
Examples of faces (Black females) from each set of faces. The first four faces—Original Face (**A**), Low-Trust Face (**B**), High-Trust Face (**C**), and Single-Race Face (**D**)—come from the Chicago Face Database ^18^, which is a publicly available database consisting of high-resolution photographs of male and female human faces of various ages and ethnicities. The last face—AI-Synthesized Face (**E**)—comes from www.thispersondoesnotexist.com, which is a publicly available database of synthetic faces that were created using a generative adversarial network trained on a large dataset of real images of human faces. More information about the sets of faces can be found in the Supplemental Materials online.

Here, we test whether facial heterogeneity affects the psychometric properties (validity, reliability, measurement equivalence) of SFT using an online survey experiment. In September of 2022, we recruited 5001 Qualtrics web panel members living in the United States. Respondents were randomly assigned to one of five conditions or sets of faces (see Fig. 1 for an illustrative example of the faces in question): (i) six “original” faces from SFT, (ii) six “low trust” faces, (iii) six “high trust” faces, (iv) six “single race” faces, and (v) six “AI-synthesized” faces. Irrespective of the condition, respondents rated each face along four trust domains: keeping a secret, repaying a loan, watching a loved one, and providing financial advice. To assess consistency across psychometric tests, we also measured respondents’ demographic characteristics (e.g., age, gender), economic preferences including positive reciprocity and unconditional altruism^16^, and prior trusting behaviors such as lending money to friends and leaving one’s door unlocked^5^.

We find that facial heterogeneity has negligible effects on the measurement validity and measurement equivalence of SFT. Tests evaluating measurement invariance, or the extent to which a latent construct is measured in the same way across groups, show that the factor loadings and item intercepts of SFT are invariant across sets of faces. Psychometric tests that assess the extent to which SFT correlates with closely related constructs with which it should correlate (convergent validity), with different constructs with which it should not correlate (discriminant validity), and with criterion variables with which it should predict (concurrent validity) yield largely consistent results across sets of faces. The one exception is the “low trust” faces. For this set of faces, we observe slight mean differences, as well as minor differences in the regression coefficients for two of the five criterion variables used to assess convergent validity. These results provide further evidence for the accuracy and consistency of SFT, and the utility of using AI-synthesized faces in social science research.

## Results

### Measurement equivalence

Nested model comparisons between configural invariance (*N* = 4753, SRMR = 0.019, RMSEA = 0.071, CFI = 0.974, TLI = 0.957), metric invariance (*N* = 4753, SRMR = 0.035, RMSEA = 0.071, CFI = 0.962, TLI = 0.957), and scalar invariance (*N* = 4753, SRMR = 0.044, RMSEA = 0.079, CFI = 0.939, TLI = 0.946) models yield inconsistent changes to SRMR, RMSEA, CFI, and TFI. These tests indicate that SFT’s factor loadings and item intercepts are invariant across sets of faces.

### Convergent validity

Table 1 shows that the effect sizes (or standardized βs) of the original SFT faces parallel previously published estimates^7,10^. Joint or familywise tests of the equality of coefficients fail to reject the null hypothesis that interaction effects are equal to zero, except for IST, *F*(4, 4650) = 3.88, *p* = 0.003, and PST, *F*(4, 4709) = 2.41, *p* = 0.046. In both cases (i.e., IST and PST), the coefficients of the “low trust” faces are statistically significantly smaller than those of most other sets of faces (Δβs $\le$ 0.10). In all other cases, the effect size differences between the coefficients are relatively trivial and statistically non-significant (see the Supplemental Materials online for coefplots showing the regression coefficients and their confidence intervals).

**Table 1:** Familywise tests of interactions between SFT and sets of faces.

	β	Familywise test
*Convergent validity*		
1ST	.711***	*F*(4, 4650) = 3.88, *p* = .003
MST	.274***	*F*(4, 4678) = 0.15, *p* = .963
GST	.424***	*F*(4, 4616) = 1.67, *p* = .153
PST	.194***	*F*(4, 4709) = 2.41, *p* = .046
POT	.413***	*F*(4, 4686) = 1.29, *p* = .217
*Discriminant validity*		
Patience	.103**	*F*(4, 4596) = 0.87, *p* = .481
Risk taking	.222***	*F*(4, 4691) = 0.97, *p* = .424
Gift exchange	.046	*F*(4, 4458) = 1.06, *p* = .376
Return a favor	− .126***	*F*(4, 4704) = 2.15, *p* = .072
Take revenge	.260***	*F*(4, 4652) = 1.92, *p* = .104
Punish unfair (self)	.111**	*F*(4, 4496) = 0.19, *p* = .941
Punish unfair (others)	.130***	*F*(4, 4453) = 0.57, *p* = .686
Donation decision	.250***	*F*(4, 4738) = 0.76, *p* = .553
Give to good cause	.009	*F*(4, 4674) = 0.89, *p* = .470
*Concurrent validity*		
Trusting behavior	.378***	*F*(4, 4720) = 0.91, *p* = .457

β = standardized beta of original SFT scale.

IST, imaginary stranger trust scale; MST, misanthropy scale; GST, generalized social trust scale; PST, particularized social trust scale; POT, political trust scale.

*p < .05, **p < .01, ***p < .001.

### Discriminant validity

Across a number of economic preferences^16^, including measures of risk preferences, time preferences, and social preferences, we find that effect sizes are statistically equivalent between the five sets of faces. For every measure of economic preferences, joint tests of the equality of coefficients fail to reject the null hypothesis that interaction effects are equal (see Table 1). As Table 1 suggests, the effect size differences between the coefficients are relatively trivial and statistically non-significant (see the Supplemental Materials online for the regression coefficients and their confidence intervals). Effect sizes of the original SFT faces replicate estimates from previous research^10^.

### Concurrent validity

The original SFT faces are moderately related to trusting behavior, β = 0.378, *p* < 0.001, supporting previous findings^7^. A joint test of the equality of coefficients fails to reject the null hypothesis that interaction effects are equal, *F*(4, 4720) = 0.91, *p* = 0.457, which is further supported by the regression coefficients and their confidence intervals (see the Supplemental Materials online).

### Descriptive analysis

**Figure 2:**
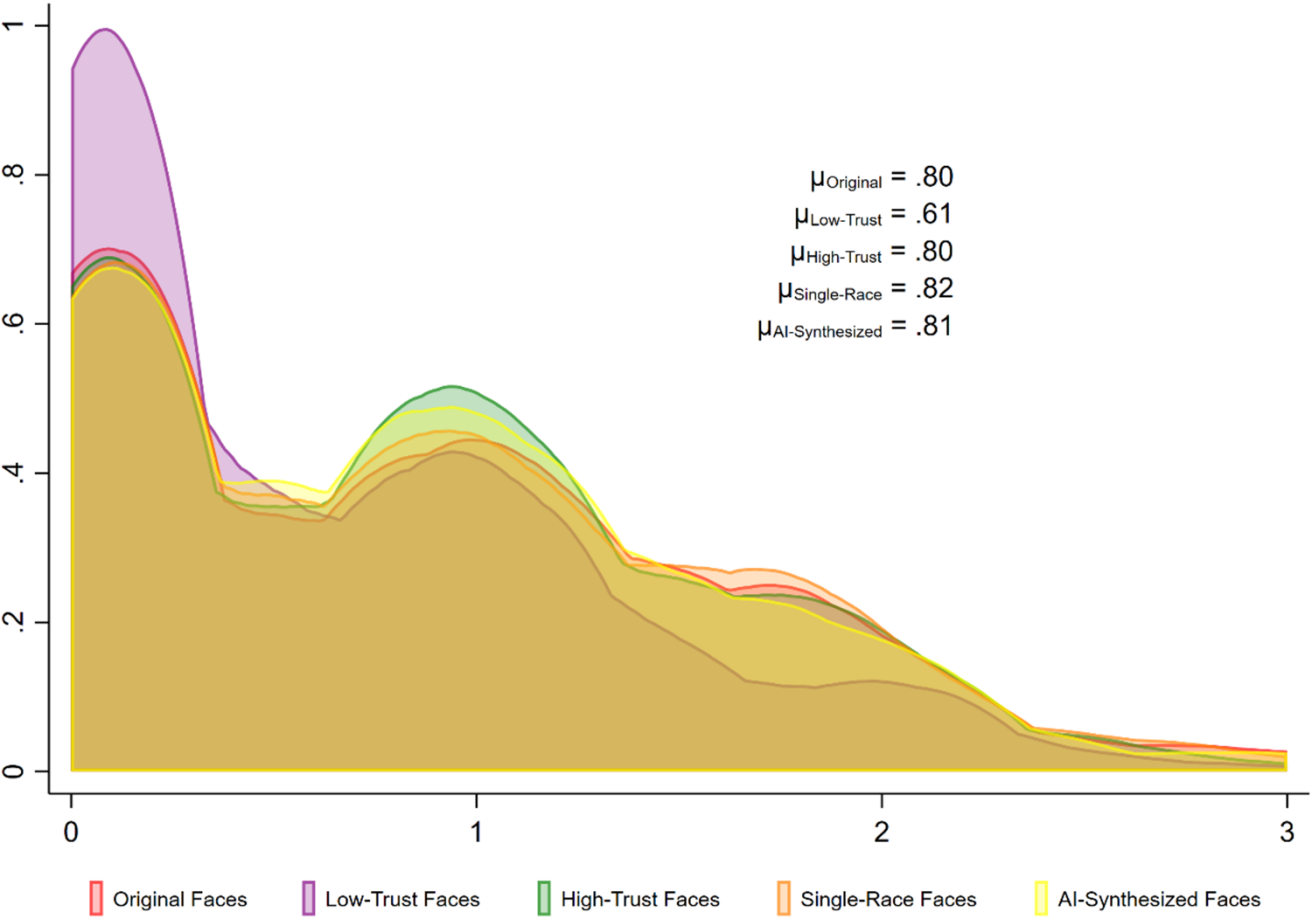
Density plots of SFT (row-mean scales) across five different sets of faces.

Figure 2 shows that the distributions of SFT across sets of faces are roughly equivalent, with the exception of low-trust faces, which have a higher density of scores below values of 1 than the other sets of faces. Row-mean scales developed by Robbins^7^ show that the mean of “low trust” faces is significantly different from all other faces, *F*(4, 4748) = 15.23, *p* < 0.001.

## Discussion and conclusion

Research shows that traditional measures of generalized trust are inaccurate and non-equivalent^4–13^. Because of these measurement issues, it is difficult to discern which scientific discoveries are real or an artifact of measurement. Some scholars have offered solutions to these longstanding measurement problems^7–10^, but the solution—SFT—rests on assumptions about the consistency of responses across different faces. We show that ratings of SFT yield reliable scores for five different sets of faces, including AI-synthesized faces, and that very little, if any, bias is introduced by manipulating the facial heterogeneity of SFT. We do, however, observe slight mean differences for “low trust” faces, as well as small differences in some tests of convergent validity, suggesting that care should be taken in selecting faces for SFT within *and* between different settings, or researchers run the risk of obtaining lower-bound estimates of generalized trust. These findings have two important implications.

First, our results extend previous research on the measurement of generalized trust^7–10^. While SFT has been shown to be more valid, reliable, and invariant than traditional measures, we additionally demonstrate the versatility and robustness of SFT to changes in its core stimuli: human faces. As an instrument, tests of factorial validity (factor loadings and item intercepts), convergent validity, discriminant validity, and concurrent validity yield comparable results regardless of the faces assessed (with the exception of a few criterion variables). These results suggest that faces can be modified in SFT without sacrificing validity or measurement equivalence. However, the extent to which this recommendation applies to other cultural contexts with different ethnoracial groups outside of the United States requires further investigation. One possible avenue for future research is to evaluate and compare the validity, reliability, and measurement equivalence of drastically different sets of faces, such as all Asian faces or all African faces, within *and* between countries that vary in their levels of ethnic heterogeneity. This would help determine which sets of faces have broader cross-cultural applicability than others.

Second, our results are consistent with recent work on synthetically generated faces, where respondents judge AI-synthesized faces and real faces as comparably trustworthy^17^. While there are growing risks associated with this technology, such as online fake profiles, fraud, and disinformation campaigns, AI-synthesized images and videos could find a home in social science research. Synthetically generated images and videos provide social scientists with opportunities to better understand human sociality without posing threats to the privacy of individuals who offer their images for purposes of research.

## Data and methods

### Sample and procedures

In September 2022, we recruited 5001 Qualtrics web panel members to participate in an online survey experiment. We included respondents who passed an attention check, lived in the United States, were 18 years of age or older, and who met our quotas for age, gender, and education. The quota sample was proportionally representative of the college-educated, U.S. population by age cohort, gender, and education. Age quotas included the following: at least 27% 18 to 34 y old, 22% 35 to 49 y old, 23% 50 to 64 y old, and 19% 65 y old plus. Gender quotas were at least 45% male and 45% female. Quotas for educational attainment were at least 8% less than high school, 25% high school diploma or equivalent, 25% some college or Associate’s degree, 20% Bachelor’s degree, and 10% graduate degree (i.e., Master’s, Doctoral, etc.). Respondents were, on average, 47.80 years old (*SD* = 17.09); 53.2% were female, 69.9% were non-Hispanic White, 43.7% had a high school diploma or equivalent, and 43.5% were married.

The survey experiment consisted of 9 blocks: some blocks were fixed at the beginning of the experiment (e.g., consent form, demographics), while the remaining blocks were presented in random order from respondent to respondent. These blocks were organized thematically, such as a block for the Imaginary Stranger Trust scale (IST), a block for SFT (where respondents were randomly assigned to one of five conditions or sets of faces), a block for traditional measures of generalized trust, a block for measures of political trust, a few blocks for measures of economic preferences, and a block for measures of trusting behavior. The survey completion rate was 33.33% (Qualtrics contacted 15,100 web panelists, and 5,001 of them completed the study). Participants who completed the study received an incentive of various types (e.g., cash, airline miles, etc.). The median length of the study was 7.43 min.

### Instruments and measures

Six different strangers were initially selected for inclusion in the “original” SFT scale^7–10^. The strangers were drawn from the Chicago Face Database^18^, which is a publicly available database consisting of high-resolution photographs of male and female human faces of various ages and ethnicities. Each face in the database is represented by a neutral expression photograph that has been normed by an independent rater sample (> 1000 independent raters). The six faces were selected based on four criteria: age (median U.S. age), race (Caucasian, African-American, and Latino/a), gender (male and female), and perceived trustworthiness (neutral ratings).

For three other sets of faces, including the “low trust”, “high trust”, and “single race” faces, we again selected faces from the Chicago Face Database, but used different selection criteria. The faces represent the genders (male and female) and races (Caucasian, African American, and Latino/a) that received the lowest perceived trustworthiness ratings (“low trust”), the highest perceived trustworthiness ratings (“high trust”), or the most consensus on perceived race (“single race”) from the independent rater sample for a given gender × race. Unlike the “original” faces, age was not a criterion for selecting these faces.

For the AI-synthesized faces, we selected images from the www.thispersondoesnotexist.com. The website uses an algorithm trained on a large dataset of real images of human faces. It then uses a generative adversarial network to fabricate faces. The authors selected the six “AI-synthesized” faces given their perceptions of gender (male and female) and race (Caucasian, African-American, and Latino/a). More information about each face can be found in the Supplemental Materials online.

For all five sets of faces, SFT asks respondents, “Imagine meeting the following stranger for the first time. Please identify how much you would trust this stranger for each of the following.” In contrast, IST, a short-form scale developed by Robbins^7^, does not show respondents faces. Instead, respondents are asked, “imagine meeting a total stranger for the first time. Please identify how much you would trust this stranger for each of the following.” SFT and IST contain four domains (or matters) of trust for which respondents would rate each of the six human faces (SFT) and the imaginary stranger (IST): (i) “To keep a secret that is damaging to your reputation” (SECRET); (ii) “To repay a loan of one thousand dollars” (LOAN); (iii) “To look after a child, family member, or loved one while you are away” (CHILD); and (iv) “To provide advice about how best to manage your money” (ADVICE). Each trust domain is measured using a 4-point scale, ranging from *Do not trust at all* to *Trust completely*, with *Do not trust very much* and *Trust somewhat* in-between the anchors, and a *Don’t know* option at the end of the scale. For SFT, the order of the six faces is randomized across respondents, meaning that for this study, respondents are randomly assigned to a set of faces and the order of the faces within each set is randomized from respondent to respondent. The order of the four trust domains is also randomized across faces. For IST, only the order of the four domains is randomized across respondents.

Convergent validity is determined by assessing the degree to which an operationalization is similar to (or converges on) other operationalizations to which it should theoretically be similar. In addition to IST, four other instruments common to the General Social Survey and the World Values Survey were used to establish convergent validity: a 3-item Misanthropy Scale (MST); a 3-item Generalized Social Trust scale (GST); a 3-item Particularized Social Trust scale (PST); and a 4-item Political Trust scale (POT). We selected these four scales because they have been used as criterion variables in previous work evaluating the validity of SFT^7,9,10^. Information on the wording and scaling of each item can be found in the Supplemental Materials online.

Discriminant validity is determined by assessing the degree to which an operationalization is not similar to (or diverges from) other operationalizations to which it should not theoretically be similar. Many theories in the social sciences assume that a set of preferences motivates behavior, such as preferences for risk, the timing of costs and benefits, and reciprocity and altruism. Recent research has shown that generalized trust is weakly correlated with several kinds of economic preferences, such as positive reciprocity and unconditional altruism^10,16^. These findings are intuitive because trust is a belief, not a preference. As a result, generalized trust should be weakly correlated with economic preferences like positive reciprocity^10^. To measure economic preferences, we used 9 items from Falk et al.^16^ that capture time preferences, risk preferences, positive and negative reciprocity, and unconditional altruism. Information on the wording and scaling of each item can be found in the Supplemental Materials online.

Concurrent validity is determined by assessing the degree to which an operationalization predicts an outcome that it should theoretically predict. Concurrent validity, in other words, identifies the strength of a relationship between the operationalization and a criterion variable at the time the operationalization is administered (or measured). Note that concurrent validity is different from predictive validity, which determines the ability of an operationalization to predict an outcome (i.e., criterion variable) in the future. By this logic, self-report measures of trust should be correlated with measures of trusting behavior, such as lending money and personal possessions to friends^5^. A 3-item scale (Trusting Behavior) adapted from Glaeser et al.^5^ was used to establish concurrent validity. Information on the wording and scaling of each item can be found in the Supplemental Materials online.

### Analysis

We investigate measurement equivalence by comparing three nested models with multiple group analysis^19^. In multiple group analysis, each successive model includes the previous model restrictions plus additional constraints. Model 1, the configural invariance model, tests the equivalence of the factor structure. Model 2, the metric invariance model, tests the equivalence of the factor loadings. Model 3, the scalar invariance model, tests the equivalence of measurement intercepts. Nested models can be tested with χ2 difference tests, but since the test is sensitive to sample size we rely on changes to tests of absolute and relative model fit.

The tests we use include the root mean squared error of approximation (RMSEA), the standardized root mean square residual (SRMR), the comparative fit index (CFI), and the Tucker-Lewis index (TLI). RMSEA and SRMR are tests of absolute model fit, while CFI and TLI are tests of relative model fit. The RMSEA is an index that measures the difference between the hypothesized model and the population covariance matrix. RMSEA ranges between 0 and 1, with values less than 0.08 indicating adequate model fit. The SRMR is a standardized measure of the square root of the difference between the sample covariance matrix and the model covariance matrix. Like the RMSEA, SRMR ranges between 0 and 1, with values less than 0.08 indicating adequate model fit. By contrast, the CFI adjusts for issues of sample size inherent to the χ^2^ test of model fit, and measures the relative improvement in model fit going from the baseline model (i.e., a model with the worst fit) to the hypothesized model. CFI ranges from 0 to 1, with values greater than 0.90 suggesting adequate model fit. The TLI measures the relative reduction in misfit per degree of freedom for the baseline model and the hypothesized model. TLI ranges from 0 to 1 but can occasionally be negative or exceed 1, with values greater than 0.90 indicating adequate model fit. We use criteria of a 0.015 change in RMSEA and SRMR paired with changes in CFI and TLI of 0.01. If nested model comparisons yield ΔRMSEA > 0.015, ΔSRMR > 0.015, ΔCFI > 0.01, *and* ΔTLI > 0.01, then the null hypothesis of equivalence should be rejected^20,21^.

We report nested model comparisons between models that estimated configural invariance, metric invariance, and scalar invariance by groups (i.e., sets of faces). To identify the configural invariance model, we follow Vandenberg and Lance^19^ and constrained the factor means and variances to 0 and 1, respectively, across groups. Factor loadings as well as item intercepts and residuals variances were freely estimated across groups. To identify the metric invariance model, we constrained the factor loadings to equality across groups, constrained the factor variance to 1 for the first group (i.e., Original Faces) but freely estimated the factor variances for all other groups, constrained the factor means to 0 across groups, and freely estimated item intercepts and residual variances across groups. To identify the scalar invariance model, we constrained the factor loadings and item intercepts to equality across groups, constrained the factor variance and mean to 1 and 0, respectively, for the first group (i.e., “original” faces) but freely estimated the factor variances and means for all other groups, and freely estimated item residual variances across groups.

For all measurement validity tests found in Table 1, we estimated the following models:

$$CriterionVariable = {\beta}_0 + {\beta}_1SFT + {\beta}_1 FaceDummies + {\beta}_3 \text (SFT \times FaceDummies) + \varepsilon$$

where *CriterionVariable* is a validation variable, such as IST or MST, SFT is a single continuous variable consisting of latent factor scores of each set of faces (Original Faces,…, AI-Synthesized Faces), *FaceDummies* is a vector of dummy variables for each set of faces (Original Faces is the referent category), and *SFT ×* *FaceDummies* is a vector of interaction effects between latent factor scores and dummy variables of each set of faces.

### Ethics approval

Ethics approval was obtained from the New York University Abu Dhabi Institutional Review Board (Approval Number: HRPP-2022-87). Our work also conforms to the Code of Ethics of the American Sociological Association (ASA), and although our study is not a medical study, we adhere to the World Medical Association’s Code of Ethics (Declaration of Helsinki) for the protection of human research participants.

### Consent to participate

Informed consent was obtained from all study participants.

### Supplementary Information

Supplementary Information.

## Data Availability

The datasets generated and/or analyzed during the current study are available in the SocArXiv repository, https://osf.io/s7uza/.
