# Supplementary material for: The effects of facial appearance on measures of generalized trust
Source: Sci Rep. 2024 Aug 8;14:18435. doi: 10.1038/s41598-024-69562-6 (PMC11310463; doi:10.1038/s41598-024-69562-6)
Supplement: Supplementary file 1 — Supplementary Information. [file 41598_2024_69562_MOESM1_ESM.pdf]

Supplemental Material

The Effects of Facial Appearance on Measures of Generalized Trust

Contents

1. Instruments and Measures
1.1. Information about each Face 2
1.2. Figure S1. Sets of Faces 3
1.3. Survey Items and Scales 4
2. Analysis
2.1. Covariate Balance 6
3. Assessments of Convergent, Discriminant, and Concurrent Validity
3.1. Figure S2. Effects of Facial Heterogeneity on Convergent Validity 7
3.2. Figure S3. Effects of Facial Heterogeneity on Convergent Validity 8
3.3. Figure S4. Effects of Facial Heterogeneity on Discriminant Validity 9
3.4. Figure S5. Effects of Facial Heterogeneity on Discriminant Validity 10
3.5. Figure S6. Effects of Facial Heterogeneity on Discriminant Validity 11
3.6. Figure S7. Effects of Facial Heterogeneity on Concurrent Validity 12

1. Instruments and Measures

1.1 Information about each Face

We use alpha numeric labels from the Chicago Face Database and www.thispersondoesnotexist.com. Recall that the AI-Synthesized Faces do not have norming data (e.g., information about age, trustworthiness).

- **Original Faces**: WF-228 (Age = 36; 96 percent White; Trustworthy = 3.88); WM-225 (Age = 36; 100 percent White; Trustworthy = 3.70); BF-214 (Age = 32; 85 percent Black; Trustworthy = 3.76); BM-021 (Age = 36; 97 percent Black; Trustworthy = 3.87); LF-240 (Age = 43; 65 percent Latina; Trustworthy = 3.75); LM-227 (Age = 32; 65 percent Latino).
- **Low-Trust Faces**: WF-240 (Age = 44; 62 percent Latina; Trustworthy = 2.42); WM-249 (Age = 43; 100 percent White; Trustworthy = 2.30); BF-200 (Age = 33; 79 percent Black; Trustworthy = 2.37); BM-219 (Age = 24; 11 percent Black; Trustworthy = 2.40); LF-218 (Age = 26; 46 percent Latina; Trustworthy = 3.75); LM-226 (Age = 37; 52 percent Latino; Trustworthy = 2.31).
- **High-Trust Faces**: WF-203 (Age = 23; 44 percent White; Trustworthy = 4.51); WM-257 (Age = 30; 85 percent White; Trustworthy = 3.92); BF-251 (Age = 38; 93 percent Black; Trustworthy = 4.62); BM-249 (Age = 19; 100 percent Black; Trustworthy = 4.51); LF-249 (Age = 28; 55 percent Latina; Trustworthy = 4.31); LM-201 (Age = 31; 7 percent Latino; Trustworthy = 4.57).
- **Single-Race Faces**: WF-207 (Age = 24; 100 percent White; Trustworthy = 3.95); WM-214 (Age = 25; 100 percent White; Trustworthy = 3.72); BF-250 (Age = 31; 100 percent Black; Trustworthy = 4.04); BM-200 (Age = 26; 100 percent Black; Trustworthy = 4.03); LF-214 (Age = 29; 100 percent Latina; Trustworthy = 3.45); LM-232 (Age = 31; 92 percent Latino; Trustworthy = 3.03).
- **AI-Synthesized Faces**: White Female (001072); White Male (002780); Black Female (001227); Black Male (003490); Latin Female (003369); Latin Male (003759).

1.2 Sets of Faces

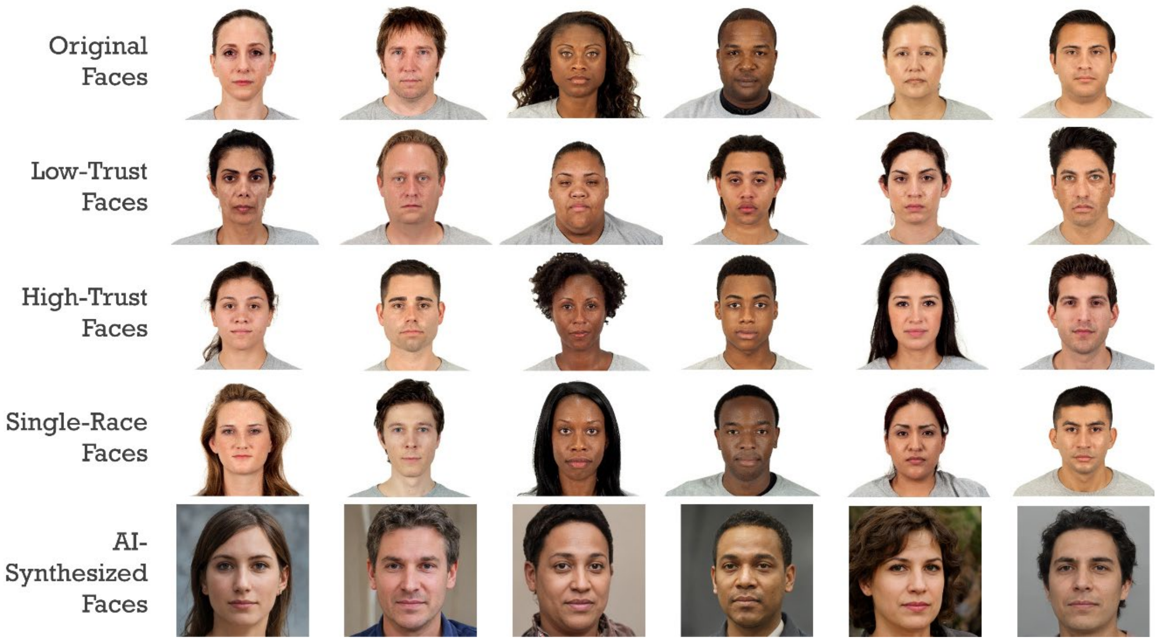

Figure S1. Five sets of faces shown randomly to respondents.
Notes: The first four sets of faces—Original Faces, Low-Trust Faces, High-Trust Faces, and Single-Race Faces—come from the Chicago Face Database (18), which is a publicly available database consisting of high-resolution photographs of male and female human faces of various ages and ethnicities. The last set of faces—AI-Synthesized Faces—comes from www.thispersondoesnotexist.com, which is a publicly available database of synthetic faces that were created using a generative adversarial network trained on a large dataset of real images of human faces.

1.3 Survey Items and Scales

Measures of convergent validity. MST consists of 3 items scored on a dichotomous answer scale. Item 1 (TRUST) asks "Generally speaking, would you say that most people can be trusted or that you need to be very careful in dealing with people?" with *Most people can be trusted* and *Need to be very careful in dealing with people* as anchors with a *Don't know* option. Item 2 (FAIR) asks "Do you think that most people would try to take advantage of you if they got the chance or would they try to be fair?" with *Most people would try to take advantage of me* and *Most people try to be fair* as anchors with a *Don't know* option. Item 3 (HELP) asks "Would you say that most of the time people try to be helpful or that they are mostly looking out for themselves?" with *People mostly try to be helpful* and *People mostly look out for themselves* as anchors with a *Don't know* option. Don't know responses were treated as system missing and all 3 items were recoded to parallel polarity (i.e., 1 = most people can be trusted, most people try to be fair, and people mostly try to be helpful).

GST consists of 3 items scored on a 4-point scale anchoring at *Do not trust at all* and *Trust completely*, with *Do not trust very much* and *Trust somewhat* in-between the anchors, and a *Don't know* option at the end of the scale. The question asked "Could you tell me for each whether you trust people from this group completely, somewhat, not very much, or not at all?" with grid items being "people you meet for the first time" (FIRST), "people of another religion" (RELI), and "people of another nationality" (NATION). Don't know responses were treated as system missing.

To measure PST, we used 3 items scored on a 4-point scale anchoring at *Do not trust at all* and *Trust completely* with *Do not trust very much* and *Trust somewhat* in-between the anchors and a *Don't know* option at the end of the scale. The question asked "Could you tell me for each whether you trust people from this group completely, somewhat, not very much or not at all?" with grid items being "your family" (FAMILY), "your neighborhood" (NEIGH), and "people you know personally" (KNOW). Don't know responses were treated as system missing.

To measure POT, we used 4 items scored on a 4-point scale anchoring at *None at all* and *A great deal*, with *Not very much* and *Quite a lot* in-between the anchors, and a *Don't know* option at the end of the scale. The question asked "We are going to list a number of organizations. For each one, could you tell us how much confidence you have in them: Is it a great deal of confidence, quite a lot of confidence, not very much confidence, or none at all?" with grid items being "the police" (POLICE), "the courts" (COURTS), "the government" (GOVERN), and "political parties" (PARTIES). Don't know responses were treated as system missing.

Measures of discriminant validity. To measure economic preferences, we used 9 items from Falk et al. (11), which capture time preferences, risk preferences, positive and negative reciprocity, and altruism. The first item, Patience, is used to measure time preferences. The measure of patience is a self-report of willingness to wait on an 11-point scale anchoring at *Completely unwilling to do so* and *Very willing to do so*, with a *Don't know* option at the end of the scale. The question asked "How willing are you to give up something that is beneficial for you today in order to benefit more from that in the future?" Risk preferences were also elicited through self-reports. The measure of risk preferences, Risk Taking, asks for the respondents' self-assessment of their willingness to take risks on an 11-point scale ("In general, how willing or unwilling are you to take risks?"). The scale anchored at *Completely unwilling to take risks* and *Very willing to take risks*, with a *Don't know* option at the end of the scale.

Positive reciprocity consists of 2 items: (i) Gift Exchange, and (ii) Return a Favor. For Gift Exchange, respondents were asked to imagine that they got lost in an unfamiliar area and that a stranger—when asked for directions—offered to take them to their destination. Respondents were then asked which out of six hypothetical presents (worth between USD $5 and $30) they would give to the stranger as a "thank

you" (with a *No present* and *Don't know* option anchored at either end of the scale). For Return a Favor, respondents were asked to provide a self-report of how willing they are to return a favor on an 11-point scale ("When someone does me a favor, I am willing to return it") anchoring at *Does not describe me at all* and *Describes me perfectly*, with a *Don't know* option at the end of the scale.

Negative reciprocity was elicited through three self-assessments: (i) Take Revenge, (ii) Punish Unfair Behavior (Self), and (3) Punish Unfair Behavior (Other). First, respondents were asked how willing they are to take revenge on an 11-point scale: "If I am treated very unjustly, I will take revenge at the first occasion, even if there is a cost to do so." The response scale was anchored at *Does not describe me at all* and *Describes me perfectly*, with a *Don't know* option at the end of the scale. The second and third items probed respondents about their willingness to punish someone for unfair behavior, either toward themselves or a third person. The question asked: "Even if there are costs for you, how willing are you to punish someone who treats the following people unfairly?", with "You" and "Others" as grid items. Both grid items anchored at *Completely unwilling to do so* and *Very willing to do so*, with a *Don't know* option at the end of the scale.

Altruism was measured with two items: (i) Donation Decision, and (ii) Give to Good Cause. For Donation Decision, respondents were asked to imagine a hypothetical situation where they unexpectedly received USD $1,000 and to state how much of this amount they would donate ("Imagine the following situation: Today you unexpectedly received $1,000. How much of this amount would you donate to a good cause?"). Respondents were allowed to enter any amount between $0 and $1,000 into a numerical box. For Give to Good Cause, respondents were asked how willing they would be to give to good causes without expecting anything in return on an 11-point scale ("How willing are you to give to good causes without expecting anything in return?"). Response options were anchored at *Completely unwilling to do so* and *Very willing to do so*, with a *Don't know* option at the end of the scale.

Measures of concurrent validity. A 3-item scale, Trusting Behavior, was used to establish concurrent validity. The instrument consists of 3 items scored on a 5-point scale ranging from *Never* to *Infrequently* to *Sometimes* to *Often* to *Very often*, with a *Don't know* option at the end of the scale. The question reads, "We would like to ask you some questions about prior behaviors. Could you tell us whether you do the following very often, often, sometimes, infrequently, or never?", with grid items for "How often do you lend personal possessions to your friends (tools, books, your car or bicycle, etc.)?", "How often do you lend money to your friends?", and "How often do you leave your door unlocked?". Don't know responses were treated as system missing. Given that measures of trusting behavior are positively correlated with traditional measures of generalized trust and with behavioral measures of trust observed in the laboratory (5), we expect similar relationships between Trusting Behavior and SFT (10).

2. Analysis

2.1 Covariate Balance

To test whether covariates are balanced across experimental conditions (i.e., sets of faces), we estimated a single multinomial logit model regressing a nominal variable for sets of faces on a vector of demographic variables, including age, gender identity, marital status, race-ethnicity, educational attainment, employment status, and U.S. region. A χ^2 test of overall model significance comparing the intercept-only model to the covariate-balance model fails to reject the null hypothesis that the two models are equal, $\chi^2(96, N = 4{,}748) = 90.29$, $p = .645$.

3. Assessments of Convergent, Discriminant, and Concurrent Validity

3.1 Effects of Facial Heterogeneity on Convergent Validity

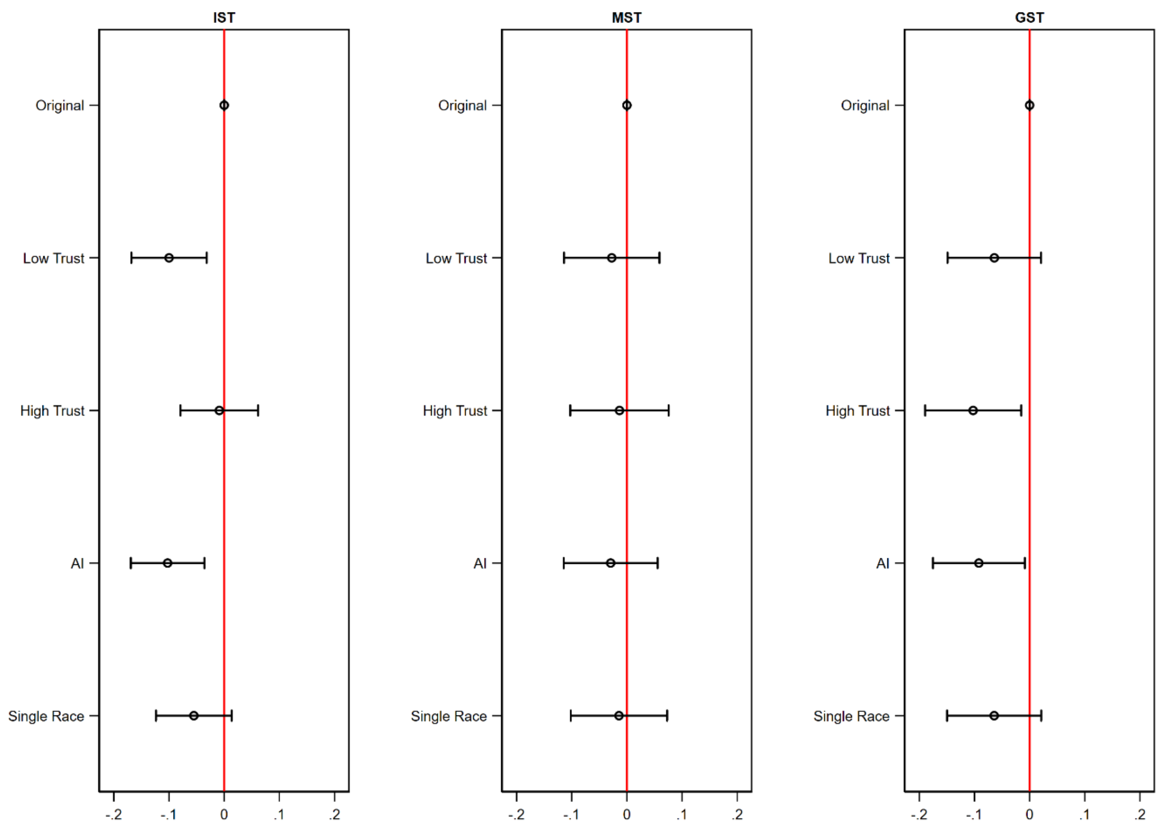

Figure S2. Standardized Coefficients of the Effects of Facial Heterogeneity on Convergent Validity

Notes: IST = Imaginary Stranger Trust scale; MST = Misanthropy Scale; GST = Generalized Social Trust scale. The interaction of SFT and the five different sets of faces (ref. = Original Faces). Bars around standardized coefficients reflect 95% CIs. Familywise tests of interaction effects between SFT and sets of faces were statistically significant for IST, $F(4, 4650) = 3.88$, $p = .003$, but not MST, $F(4, 4678) = 0.15$, $p = .963$, or GST, $F(4, 4616) = 1.67$, $p = .153$.

3.2 Effects of Facial Heterogeneity on Convergent Validity

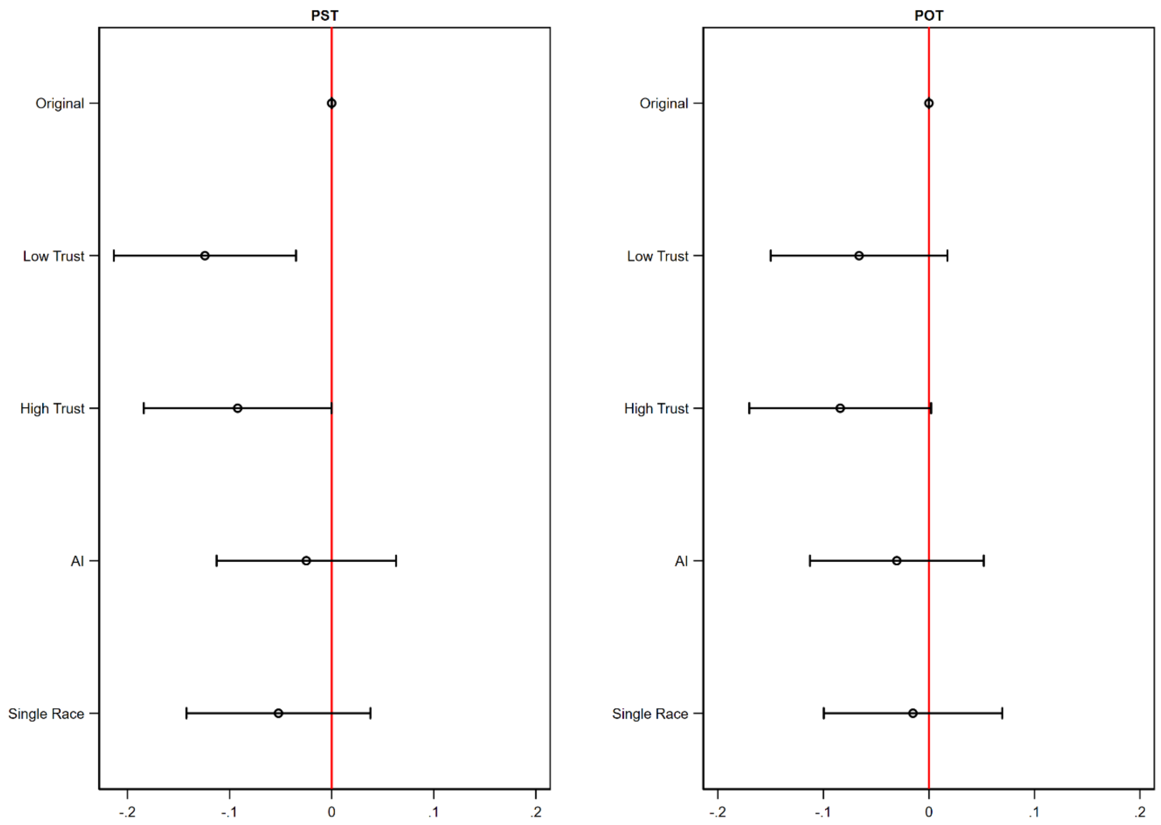

Figure S3. Standardized Coefficients of the Effects of Facial Heterogeneity on Convergent Validity

Notes: PST = Particularized Social Trust scale; POT = Political Trust scale. The interaction of SFT and the five different sets of faces (ref. = Original Faces). Bars around standardized coefficients reflect 95% CIs. Familywise tests of interaction effects between SFT and sets of faces were statistically significant for PST, $F(4, 4709) = 2.41$, $p = .046$, but not POT, $F(4, 4686) = 1.29$, $p = .217$.

3.3 Effects of Facial Heterogeneity on Discriminant Validity

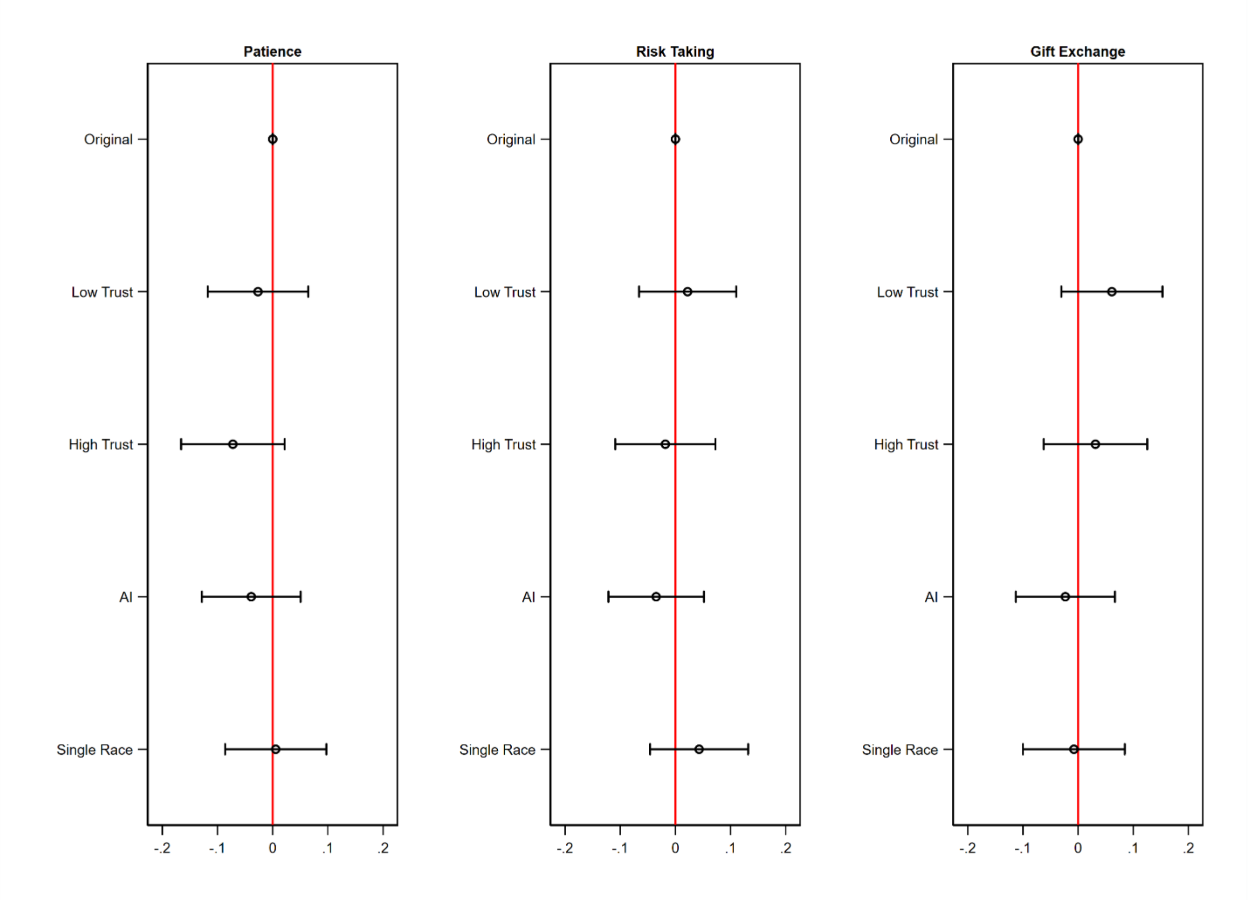

Figure S4. Standardized Coefficients of the Effects of Facial Heterogeneity on Discriminant Validity

Notes: The interaction of SFT and the five different sets of faces (ref. = Original Faces). Bars around standardized coefficients reflect 95% CIs. Familywise tests of interaction effects between SFT and sets of faces were statistically non-significant for all models. Patience: $F(4, 4596) = 0.87$, $p = .481$; Risk Taking: $F(4, 4691) = 0.97$, $p = .424$; and Gift Exchange: $F(4, 4458) = 1.06$, $p = .376$.

3.4 Effects of Facial Heterogeneity on Discriminant Validity

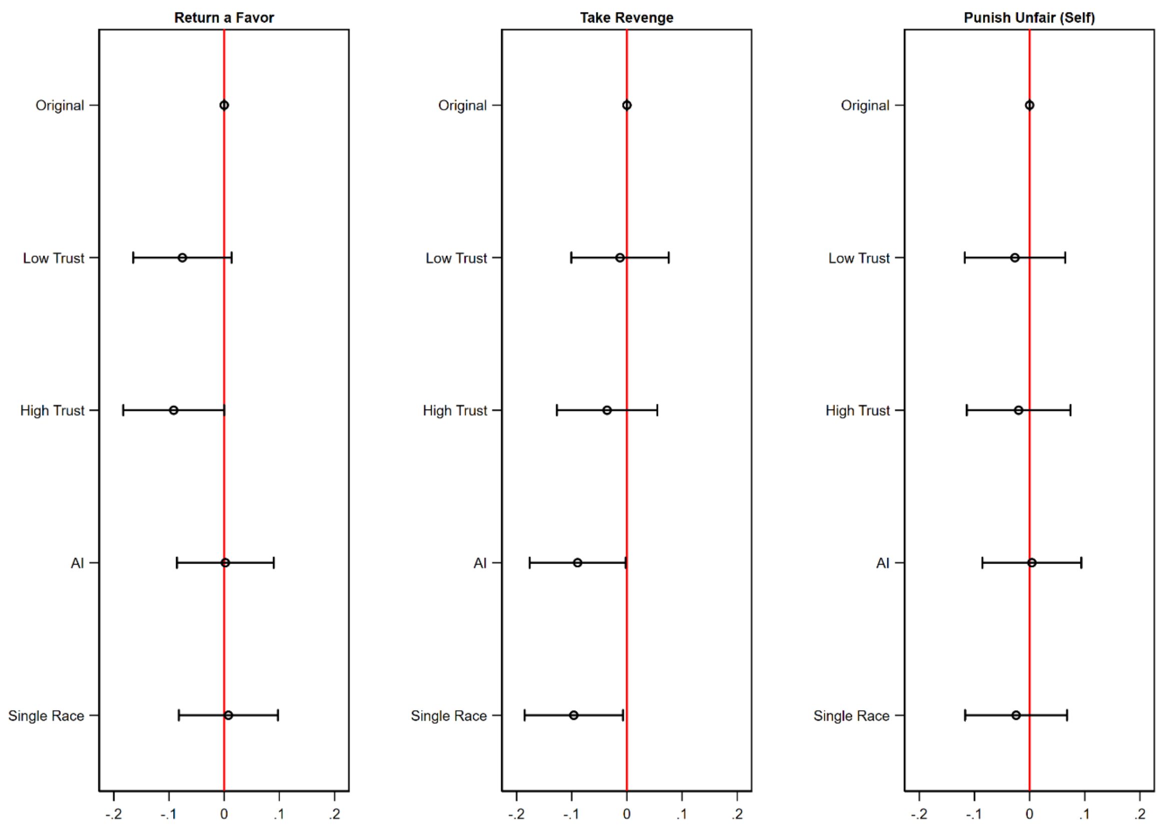

Figure S5. Standardized Coefficients of the Effects of Facial Heterogeneity on Discriminant Validity
Notes: The interaction of SFT and the five different sets of faces (ref. = Original Faces). Bars around standardized coefficients reflect 95% CIs. Familywise tests of interaction effects between SFT and sets of faces were statistically non-significant for all models. Return a favor: $F(4, 4704) = 2.15$, $p = .072$; Take Revenge: $F(4, 4652) = 1.92$, $p = .104$; and Punish Unfair (Self): $F(4, 4496) = 0.19$, $p = .941$.

3.5 Effects of Facial Heterogeneity on Discriminant Validity

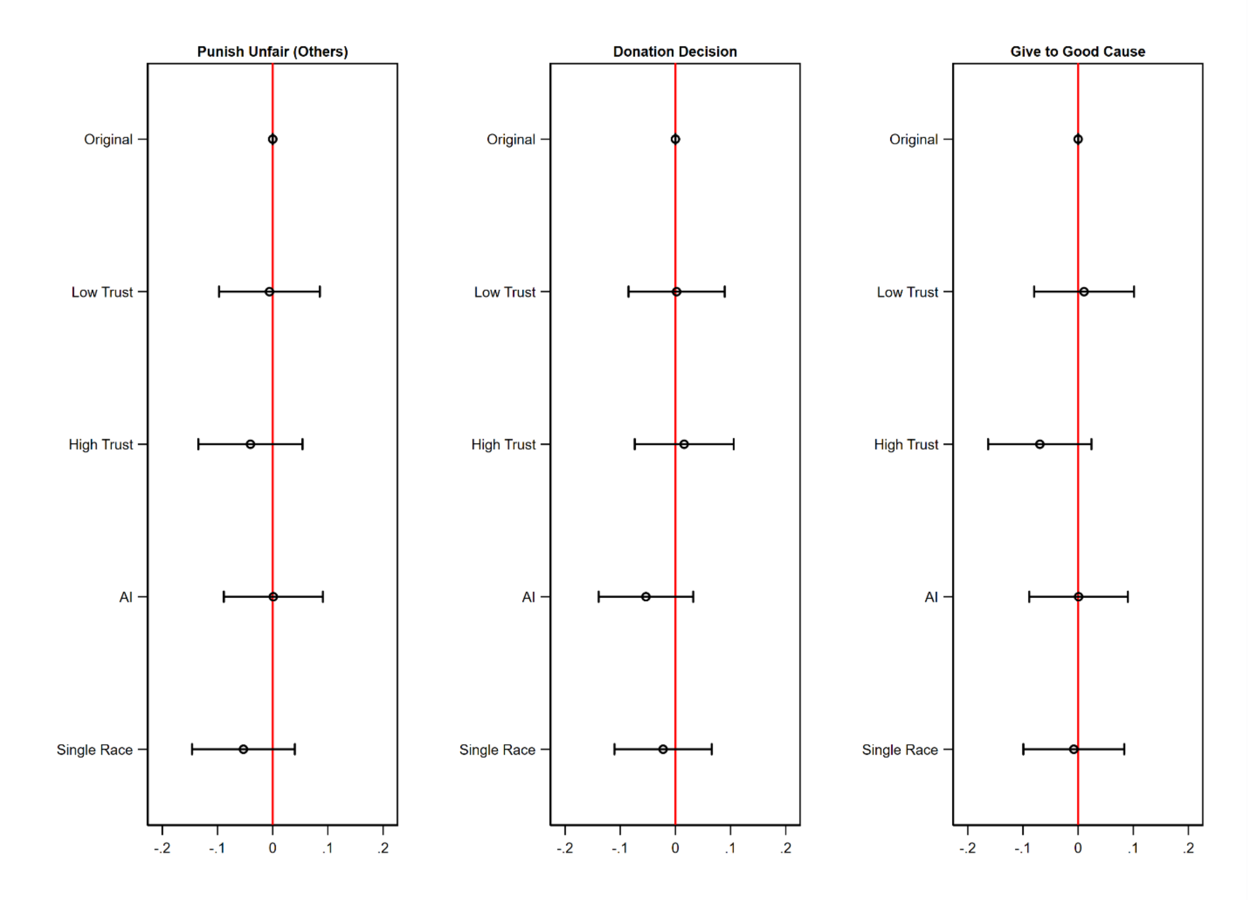

Figure S6. Standardized Coefficients of the Effects of Facial Heterogeneity on Discriminant Validity

Notes: The interaction of SFT and the five different sets of faces (ref. = Original Faces). Bars around standardized coefficients reflect 95% CIs. Familywise tests of interaction effects between SFT and sets of faces were statistically non-significant for all models. Punish Unfair (Others): $F(4, 4453) = 0.57$, $p = .686$; Donation Decision: $F(4, 4738) = 0.76$, $p = .553$; and Give to Good Cause: $F(4, 4674) = 0.89$, $p = .470$.

3.6 Effects of Facial Heterogeneity on Concurrent Validity

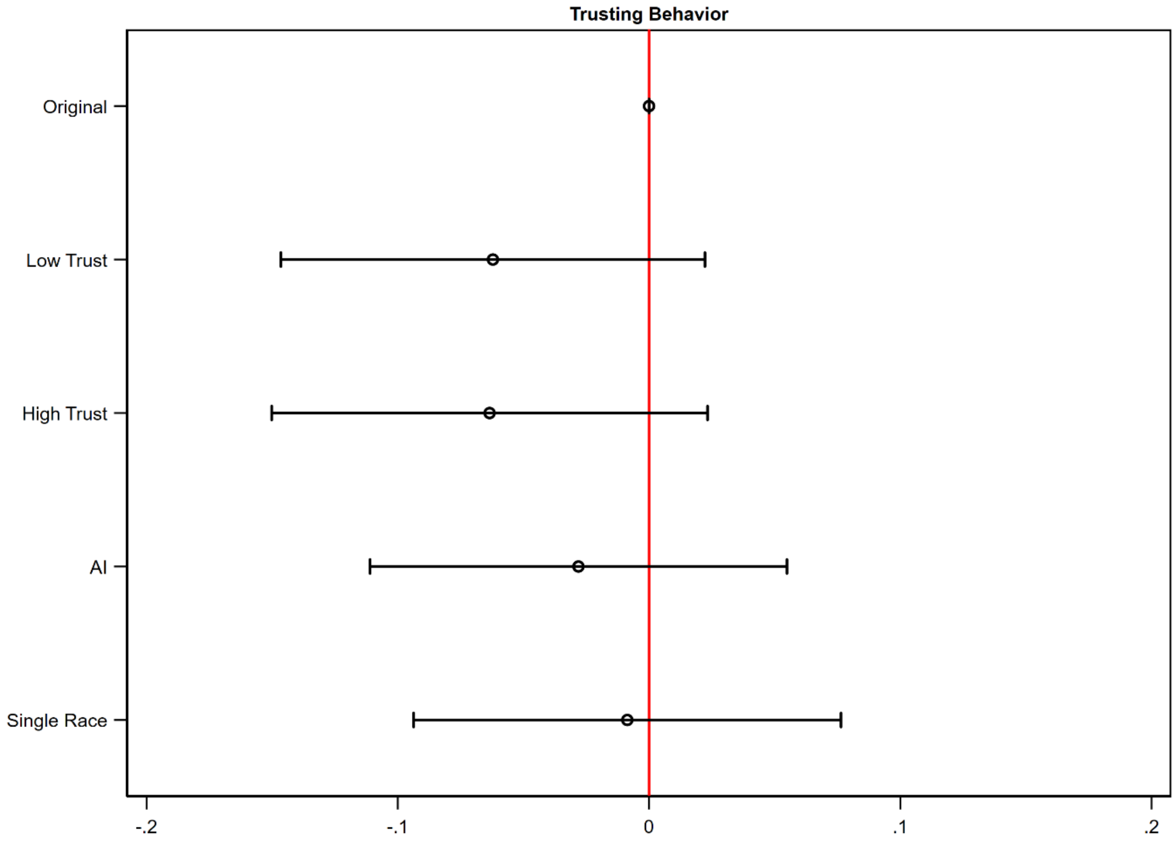

Figure S7. Standardized Coefficients of the Effects of Facial Heterogeneity on Concurrent Validity
Notes: The interaction of SFT and the five different sets of faces (ref. = Original Faces). Bars around standardized coefficients reflect 95% CIs. A familywise test of interaction effects between SFT and sets of faces was statistically non-significant for Trusting Behavior, $F(4, 4720) = 0.91$, $p = .457$.
